# Mesenchymal stem cell-derived exosomes: a new therapeutic approach to osteoarthritis?

**DOI:** 10.1186/s13287-019-1445-0

**Published:** 2019-11-21

**Authors:** Elaheh Mianehsaz, Hamid Reza Mirzaei, Maryam Mahjoubin-Tehran, Alireza Rezaee, Roxana Sahebnasagh, Mohammad Hossein Pourhanifeh, Hamed Mirzaei, Michael R. Hamblin

**Affiliations:** 1 Department of Physical Medicine and Rehabilitation, Faculty of Medicine, Kashan University of Medical Sciences, Kashan, Iran; 2 Trauma Research Center, Kashan University of Medical Sciences, Kashan, Iran; 3 Department of Medical Immunology, School of Medicine, Tehran University of Medical Sciences, Tehran, Iran; 4 Student Research Committee, Mashhad University of Medical Sciences, Mashhad, Iran; 5 Department of Medical Biotechnology, Faculty of Medicine, Mashhad University of Medical Sciences, Mashhad, Iran; 6 Department of Molecular Medicine, School of Advanced Technologies in Medicine, Tehran University of Medical Sciences, Tehran, Iran; 7 Halal Research Center of IRI, FDA, Tehran, Iran; 8 Research Center for Biochemistry and Nutrition in Metabolic Diseases, Institute for Basic Sciences, Kashan University of Medical Sciences, Kashan, I.R Iran; 9 Wellman Center for Photomedicine, Massachusetts General Hospital, Harvard Medical School, 40 Blossom Street, Boston, MA 02114 USA

**Keywords:** Osteoarthritis, Mesenchymal stem cells, Exosomes, Inflammation, Chondrocytes, Cartilage degradation, Paracrine mediators

## Abstract

Degenerative disorders of joints, especially osteoarthritis (OA), result in persistent pain and disability and high costs to society. Nevertheless, the molecular mechanisms of OA have not yet been fully explained. OA is characterized by destruction of cartilage and loss of extracellular matrix (ECM). It is generally agreed that there is an association between pro-inflammatory cytokines and the development of OA. There is increased expression of matrix metalloproteinase (MMP) and “a disintegrin and metalloproteinase with thrombospondin motifs” (ADAMTS). Mesenchymal stem cells (MSCs) have been explored as a new treatment for OA during the last decade. It has been suggested that paracrine secretion of trophic factors, in which exosomes have a crucial role, contributes to the mechanism of MSC-based treatment of OA. The paracrine secretion of exosomes may play a role in the repair of joint tissue as well as MSC-based treatments for other disorders. Exosomes isolated from various stem cells may contribute to tissue regeneration in the heart, limbs, skin, and other tissues. Recent studies have indicated that exosomes (or similar particles) derived from MSCs may suppress OA development. Herein, for first time, we summarize the recent findings of studies on various exosomes derived from MSCs and their effectiveness in the treatment of OA. Moreover, we highlight the likely mechanisms of actions of exosomes in OA.

## Introduction

Osteoarthritis (OA), one of the most common musculoskeletal diseases, is characterized by sub-chondral bone sclerosis, synovial inflammation, cartilage degradation, ligament calcification, and osteophyte formation [1, 2]. Damage to joints following trauma, obesity, age, and genetic background are the major risk factors for OA [3]. OA leads to extensive public health costs for treatment and prevention, and a considerable loss of economic productivity due to worker incapacity [4, 5]. The direct cost of OA in the Canada, USA, UK, Australia, and France accounts for 1–2.5% of the gross national product of country; however, the indirect cost of OA are much higher, and it seems the true cost of OA is underestimated [5]. Direct costs are accounted for hospital stays, surgery, diagnosis, health professional visits, and treatments [5]. The indirect costs are those paid and unpaid activities, like employment, schooling, and homemaking, that result from disability associated with the health status [5]. Since the prevalence of OA rising with age, in an aging population, the OA is a growing source of indirect costs [5].

Currently, the treatments are mainly directed to relieving the symptoms of OA using non-steroid anti-inflammatory drugs (NSAID) and painkillers [6]. Because the mentioned drugs are not very effective, cannot ameliorate the disease and its symptoms for long periods of time, and also have many side effects [7], the search for novel therapeutic strategies is an important issue [8].

Mesenchymal stem/stromal cell (MSC)-based therapy is a promising new approach for OA, which has been further developed in recent years [9–13]. Preclinical studies have indicated that the cartilage of the joint can be protected from degeneration, and the development of OA can be delayed through intra-articular injection of MSCs isolated either from adipose tissue or from bone marrow [14–17]. Furthermore, some clinical trials have evaluated MSC-based treatments for OA and have shown decreases in inflammation and pain [12]. In a phase I–II clinical trial, Soler and colleagues have utilized ex vivo expanded autologous mesenchymal stromal cells for the treatment of osteoarthritis of the knee aiming to assess their safety and cartilage regeneration capacity. Their results showed that this cell-based therapy was well tolerated, though some adverse effect events (e.g., mild arthralgia and low back pain) were reported. There was a significant decrease in the intensity of pain since day 8 after the cell product administration and that was lasted after 12 months. The SF-36 QoL assay exhibited improvement of factors including bodily pain and physical functioning at month 12. The health assessment questionnaire showed a substantial decrease of disability. T2 mapping of cartilage also revealed signs of tissue regeneration in all patients at 12 months post-therapy [18]. In another study, Matas et al. have evaluated the safety and therapeutic efficacy of the single or repeated intraarticular administration of umbilical cord-derived (UC) MSCs in the knee of OA patients(a phase I/II trial) [19]. No severe adverse events were observed. MSC-treated patients showed more significant pain and greater function improvement from baseline. At 12 months, based on Western Ontario and Mc Master Universities Arthritis Index, MSC-treated patients (MSC-2 group) significantly experienced lower levels of pain versus the hyaluronic acid (HA) group. Visual analog scale for the pain and total Western Ontario and McMaster Universities Osteoarthritis Index (WOMAC) were also considerably lower in the MSC-2 group compared with the HA group at 12 months post-therapy [19].

MSCs may secrete mediators with chondro-protective and anti-inflammatory activities contributing to therapeutic impacts [20, 21].

MSC-derived extracellular vesicles (EVs) are a mixed population of heterogeneous membranous vesicles with distinct content which are critical mediators of intercellularcommunication. These vesicles establish a vesicle-mediated transport system to regulate a wide range of biological and pathological processes [22]. They are involved in cell-to-cell communication pathways [23]. The three major categories of EVs are (a) apoptotic bodies, (b) microparticles or microvesicles, and (c) exosomes or nanovesicles [24]. EVs are specified by their dimensions, expression of membrane markers, and their biogenesis. The endosomal compartments in multivesicular bodies are involved in producing exosomes. Endosomal markers (CD9, CD61, CD83, ALIX, TSG101) are expressed by exosomes, whereas microparticles (MPs) are released by a cell membrane budding process. It should be noted that MPs express markers according to their parental cells [2].

The crucial role of MSC-derived exosomes for the regulation of cell migration, proliferation, differentiation, and extracellular matrix synthesis has been increasingly supported by recent findings [25–27]. A report in 2010 showed that exosomes are secreted as active factors by MSCs responding to damage caused by myocardial ischemia reperfusion (I/R) [28]. Moreover, reports have demonstrated that MSC exosomes contribute to the repair and regeneration of cartilage via regulating immune reactivity, diminishing apoptosis, and increasing proliferation [29–31]. Exosomes, which function as intercellular communication vehicles, are small lipid-bilayer membrane vesicles between 50 and 150 nm in diameter [32]. Exosomes are able to transfer cargos of nucleic acids (mRNAs and microRNAs), proteins, and bioactive lipids [32]. Exosomes can produce biological responses in recipient cells [28]. Some findings have shown ambiguous effects of exosomes on the immune response or possible tumorigenicity, which may be considered unfavorable properties of exosomes [33, 34]. Nevertheless, there have been few studies conducted to investigate the precise molecular mechanisms by which MSC exosomes can promote cartilage chondrogenesis [27, 31, 35–37].

Recently, numerous investigations have been carried out to evaluate the potential role of exosomes derived from stem cells isolated from different sources. An in vivo study revealed that symptoms of OA could be decreased by exosomes isolated from iPS-derived MSCs, or from synovial MSCs; however, the highest efficiency was seen for exosomes from iPS-derived MSCs [38]. However in some studies, stem cells did not have a good therapeutic benefit in the treatment of OA. Tao and colleagues showed that the anabolic activity of chondrocytes was decreased by MSC-derived exosomes, unless the MSCs had been engineered to express miR-140-5p [39]. Also, the beneficial effects of embryonic stem cell-derived exosomes have been reported in a model involving destabilizing the medial meniscus (DMM) [36]. As yet, there is no information about the effect of other kinds of EVs on OA. In this review, we summarize the pathogenic roles of exosomes in OA, and the evidence for exosomes derived from MSCs in the treatment of OA.

## Exosomes: biogenesis, cargos, and different subtypes

### Biogenesis of exosomes

**Fig. 1:**
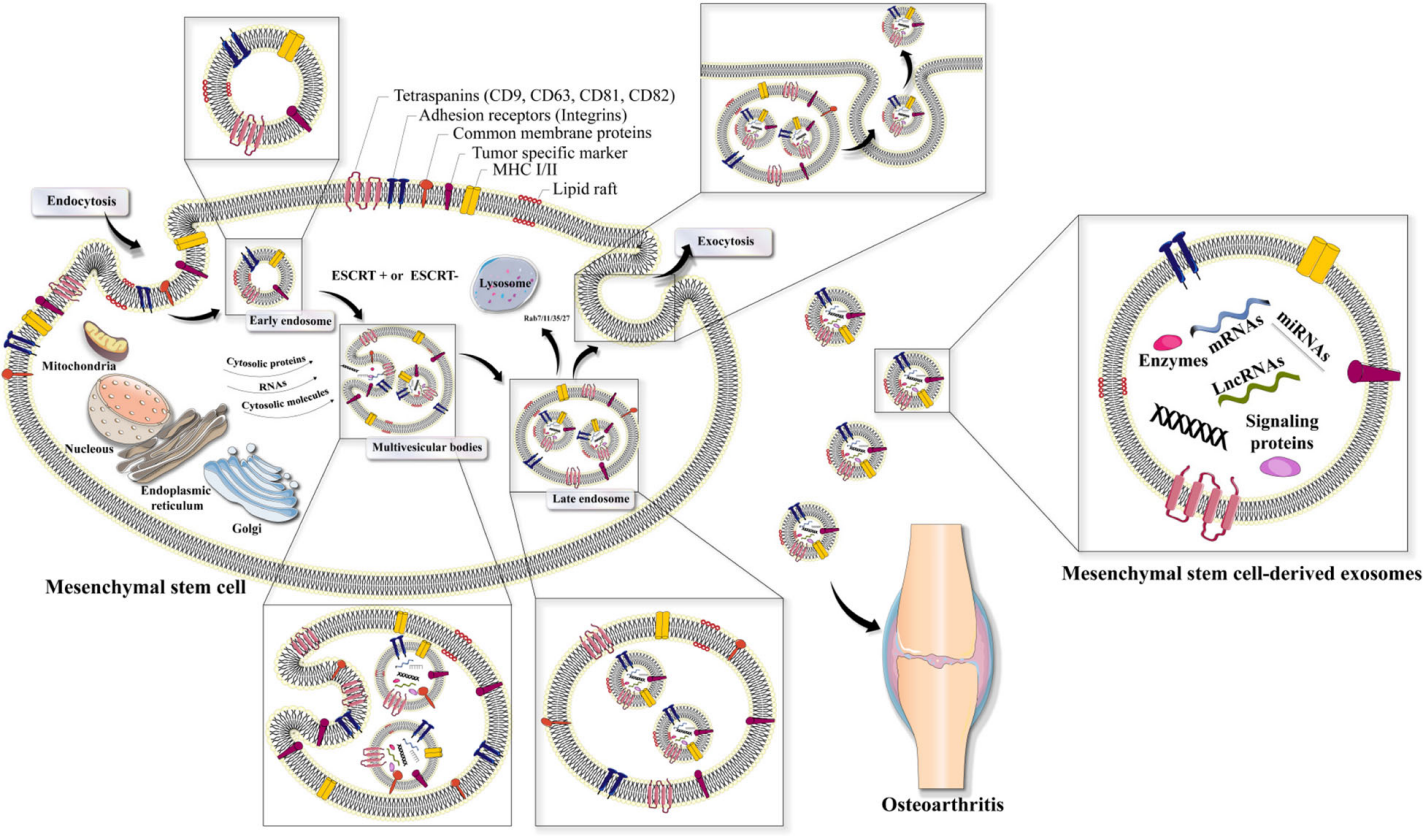
Exosome biogenesis and its relationship with osteoarthritis. A clathrin-dependent pathway or a clathrin-independent pathway initially mediates endocytosis, at a lipid raft. The endocytic vesicles contain signaling proteins, growth factor receptors, oncoproteins, combined with normal membrane proteins, including tetraspanins (e.g., CD9, CD63, and CD81), MHC I and II, and adhesion molecules (e.g., cadherins, integrins). Exosome biogenesis occurs via the endosomal network in the endosomal sorting complexes needed for ESCRT-independent or ESCRT-dependent pathways. Inward budding of MVB produces intra-luminal vesicles (exosomes). Several cytoplasmic molecules (e.g., heat shock proteins, ubiquitin-related proteins, mRNAs, microRNAs [miRNAs], cytoskeleton proteins) and nuclear molecules (e.g., long-noncoding RNAs [lncRNAs], transcriptional factors, DNAs) can be loaded into MVB by stage-specific pathways, some of which are osteoarthritis type-specific. Moreover, plasma membrane fusion of multi-vesicular bodies leads to release of exosomes by exocytosis. Numerous Rab GTPases (such as Rab11/35, Rab7, and Rab27) are present in secreted exosomes. Eventually, MSC-derived exosomes are transported to the osteoarthritis micro-environment where they modulate osteoarthritis. ESCRT, endosomal sorting complexes required for transport; MHC, major histocompatibility complex; MSCs, mesenchymal stem cells; MVB, multi-vesicular bodies; rER, rough endoplasmic reticulum; sER, smooth endoplasmic reticulum; Rab, Ras-associated binding

Exosomes have dimensions ranging between 30 and 150 nm. They are nanoscale EVs that are produced by nearly all types of cells [40, 41]. Exosomes are produced by the endosomal network, via intra-luminal vesicles (ILV, also known as pre-exosomes) formed by inward budding of the membranes of multivesicular bodies [40, 42]. Numerous different pathways [43], such as ESCRT (endosomal sorting complexes required for transport), and ESCRT-independent pathways are involved in exosome biogenesis (Fig. 1). Exosome biogenesis requires four separate multiprotein sub-complexes (ESCRT-0, ESCRT-I, ESCRT-II, and ESCRT-III) that compose the ESCRT membrane-scission machinery. Ubiquitinated proteins are bound in initial ESCRT complexes (ESCRT-0, ESCRT-I, and ESCRT-III) via their ubiquitin-binding sub-units, which result in the formation of strongly bound complexes with proteins in the cytoplasm. The ESCRT-III complex temporarily assembles on the endosome membrane and undergoes vesicle scission [44]. It has been found that additional components, such as apoptosis-linked gene 2 (ALG-2)-interacting protein X (ALIX) (a cytoplasmic protein concentrated and expressed in exosomes and phagosomes), ATPase, and vacuolar protein sorting-associated protein (VPS4) are involved in regulating the ESCRT membrane-scission machinery [44]. Moreover, lipids such as the sphingolipids, ceramide [45, 46], and sphingosine 1-phosphate function in the ESCRT-independent pathway to regulate the release of exosomes [47].

### Cargos of exosomes

The molecular components of exosomes, including lipids, nucleic acids, metabolites, and proteins, differ from each other, based on the cellular origin, environment, developmental phase, epigenetic modification, and the precise biogenesis mechanism. Moreover, studies have identified a variety of RNA types, such as microRNAs (miRNAs), messenger RNAs (mRNAs), transfer RNAs (tRNAs), long noncoding RNAs (lncRNAs), or ribosomal RNAs (rRNAs) that all can be present in exosomes [48]. Different types of RNA cargo may be involved in epigenetic modification of cells and altering biological activities. Recent studies have indicated that double-stranded DNAs (dsDNAs) found in tumor-derived exosomes could reflect the mutational status of their parental tumors [49]. Studies have shown that > 10 kb pieces of cellular genomic DNA are indicative of mutations in oncogenes or tumor suppressor genes in tumor-derived exosomes [50]. Furthermore, various bioactive proteins, which originate from the plasma membrane and the cytoplasm, exist within exosomes. Certain proteins, including Ras-related GTP-binding protein (Rab), ALIX, or ESCRT complexes that participate in exosome biogenesis, have been found in exosomes [44]. Additional proteins, including tetraspanins (i.e., CD9, CD63, and CD81), heat shock proteins (e.g., HSP70 or HSP 90), and integrins, can be packaged into exosomes, which are involved in intracellular assemblage or trafficking [40]. The expression patterns of proteins which reflect the pathophysiological status of the parental cells can be determined by analysis of exosomal proteins. The composition of the membranes of exosomes is characteristic of lipid rafts. They contain larger amounts of particular lipid species, such as sphingomyelin, phosphatidylcholine, cholesterol, ceramide, and diacylglycerol in comparison with their parental cells [51]. The release and formation of exosomes are regulated by lipid-metabolizing enzymes, such as phospholipase D2 (PLD2) or neutral sphingomyelinase (nSMase) [45].

### Subtypes of exosomes

Most researchers, who have investigated exosome-mediated cell functions, have used heterogeneous exosome preparations with different molecular cargos, and varying dimensions and morphology, all resulting in different biophysical properties. These have included large exosome vesicles (90 to 120 nm), small exosome vesicles (60 to 80 nm), and non-membranous nanoparticles (also known as exomeres, size about 35 nm) [52]. Glycomic, lipidomic, and proteomic analyses have shown different molecular profiles inside exosomes and exomeres. Proteomic analysis has shown a higher expression of enzymes associated with metabolism, hypoxia, and microtubule and coagulation-related proteins in exomeres, in comparison with large or small membranous exosomal vesicles. On the other hand, large and small exosomal vesicles contain higher amounts of signaling proteins associated with the mitotic spindle, interleukin (IL)-2/STAT5, and proteins associated with endosome secretion pathways [52]. Differences between exomeres and exosomes also involve sialylated glycoproteins (i.e., galectin-3-binding protein) [52]. Because there are differences in the lipid composition between different exosomes (and also exomeres) [52], density gradient centrifugation can separate them into two distinguishable populations: higher density exosomes (HD-Exo) and lower density exosomes (LD-Exo) [53]. An increase in “solute carrier family 38 member 1” (SLC38A1) was demonstrated in LD-Exo-treated endothelial cells in comparison with HD-Exo-treated endothelial cells [53]. Overall, studies have indicated that different biological effects can be triggered on recipient cells by using different exosomal sub-populations.

### Exosomes and osteoarthritis

mRNAs, proteins, long noncoding RNAs (lncRNA, cirRNA), and miRNAs are all found in the exosomal contents. Changes in gene expression, downstream function, and processes related to physiology or pathology may be caused by the action of exosomes on recipient cells [54].

Recently, some studies have been carried out to evaluate the various effects of exosomes on different cells involved in the joint diseases [23, 42, 55, 56]. Cell-derived EVs have been isolated from synovial fibroblasts (SF) extracted from the inflamed joints of OA and RA patients and investigated their role in the cellular processes such as inflammation and cartilage degeneration which are implicated in the disease progression [56–58]. Domenis et al. explored the immune regulatory properties of SF-derived exosomes from end-stage OA patients, on macrophages differentiated from human peripheral blood mononuclear cells (PBMCs) [59]. When patient cells were treated by exosomes, it was demonstrated that the macrophages generated a spectrum of chemokines and pro-inflammatory cytokines, such as CCL8, IL-1β, MMP12, CCL15, MMP7, and CCL20, which would result in cartilage degradation and inflammation in joints [59]. Kolhe et al. performed similar experiments and showed a significant decrease in cell survival and the expression of anabolic genes (COL2A1, ACAN), and an increase in the expression of catabolic and inflammatory genes (IL-6, TNF-α) using articular chondrocytes treated with exosomes derived from SF from OA patients [60]. Kato et al. [61] investigated whether exosomes mediated the interaction between articular chondrocytes and inflammatory synovial fibroblasts (SFBs). Exosomes were isolated from untreated similar fragment pairs (SFBs) and from similar fragment blocks (SFPs) that had been treated or not with IL-1β, and were then added to normal articular chondrocytes. These workers showed upregulation of the expression of MMP-13 and ADAMTS-5 and downregulation of ACAN and COL2A1 in articular chondrocytes when treated with IL-1β-treated SFB-derived exosomes, compared to exosomes from untreated SFBs. Additionally, exosomes from IL-1β-treated SFBs produced OA-like changes in both in vitro and in vivo models.

Two studies carried out comparisons between exosomes from various sources for discovering arthritis mechanisms. SF exosomes from patients with RA and OA were compared by Zhang et al. They observed a membrane-bound form of TNF-α in exosomes generated from SF of patients with RA; however, they did not see this phenomenon in the case of OA. Moreover, another research found that this kind of TNF-α enhances SFB exosome generation in RA and forms a positive amplification loop in RA pathogenesis [62].

Kolhe et al. compared SF-derived exosomes isolated from females and males with OA and non-OA in order to examine sex differences in OA pathophysiology. They observed a difference in the miRNA contents of EVs between groups with OA and non-OA, as well as between the two sexes [60]. The “Kyoto Encyclopedia of Genes and Genomes” (KEGG) represents a comprehensive database which is used to map associated genes to their respective pathways [63]. Though pathway maps, the KEGG pathway database provides pathway diagrams in both healthy and diseased states [64]. KEGG annotation analysis was used to assess the relationship between glycan degradation, cell adhesion molecules, and mucin type O-glycan biosynthesis and downregulated miRNAs in OA for each sex. Moreover, the possible involvement of thyroid hormone synthesis, biotin metabolism, and signaling related to amphetamine addiction was investigated along with upregulated miRNAs. Contributions from ovarian steroidogenesis and estrogen signaling pathways were also detected using KEGG annotation analysis in female OA patients [60]. Table 1 shows exosomes and their cargos involved in arthritis.

**Table 1:** Exosomes and their cargos in arthritis

Disease	Composition of exosome cargo	Model	Sample (*n*)	Function(s)	Ref
RA, OS	Fn (α, β chain), *FGB,* fibrinogen D fragment, Spα, α2M, IgG1 γ-chain C region, Fn/IgG complex	Human	15	Induction of T cells, *enhancement of T cell immunity*, Inhibition of TNF, antigen presentation	[65]
RA	Integrin CD41	Human	Not mentioned	–	[66]
RA	LYVE-1 ↑	Human	60	Lymphangiogenesis	[67]
	AA			AA tended to be higher in patients with higher disease activity	
RA	*TNF*-α	Human	10	Induction of apoptosis resistance in CD^4+^ T cell via sustaining the activities of both Akt and NF-κB, and blocking some effects of caspase activation	[62]
Collagen-induced arthritis	CD71, HSP70	Mice	2	Immunosuppressive and anti-inflammatory effects	[68]
RA	MHC I and II molecules, CD11c, CD86, CD178	Mice	–	Immunosuppressive and anti-inflammatory effects	[69]
Collagen-induced arthritis	HSC70, CD81, CD80/86, MHC I, and MHC II	Mice	2	Immunosuppressive and anti-inflammatory effects	[70]
RA, PsA	Rank in PA↓, in RA↑	Human	22	Modulation of the osteoclastogenesis	[71]
OA	hsa-*miR*-*4717*-*5p ↑*	Human	6	Modulation of the RGS2	[72]
RA	miR-17 ↑	Human	25	Regulation of Treg differentiation by inhibiting TGFBR II expression and disrupting the homeostasis of Tregs.	[73]
RA	miR-6089 ↓	Human	76	Regulation of IL-6, IL-29, and TNF-α production by targeting LPS/TLR4-mediated inflammatory response	[74]
RA	miR-548a-3p ↓	Human	76	Inhibition of proliferation and activation by regulating the TLR4/NF-κB signaling pathway	[75]
OS	Different types	Human	41	Estrogen responsive in female osteoarthritis and targeting TLR signaling pathways	[60]
RA	miR-221-3p ↑	Mice	30	Inhibition of osteoblast differentiation by suppressing the expression of Dkk2 and regulation of signaling pathways at erosion sites that affect bone loss and compensatory bone formation	[76]
OA	PCGEM1 ↑	Human	42	Triggering proliferation of osteoarthritic synoviocytes and progression of OA	[77]
RA	Hotair↑	Human	28	Activation of MMP-2 and MMP-13 in osteoclasts and RA synoviocytes and leading to migration of active macrophage	[78]
	LUST↑				
	anti-NOS2A↑				
	MEG9↑				
	TUG1↑				
	NEAT1↑				
	SNHG4↑				
	Malat1↓				
	SNHG1↓				
	PR antisense↓				
	mascRNA↓				
	PRINS↓				
	transcripts↓				
	HOXA3as↓				
OA	Not mentioned	Human	10	Stimulation of M1 macrophages to release IL-1*β/* IL-16, CCL20/CCL15/CXCL1) and MMP12/MMP7	[59]

*AA* amyloid A, *anti-NOS2A* anti-nitric oxide synthase 2 A, *CCL20* chemokine (C-C motif) ligand 20, *CD41* cluster of differentiation 41, *Dkk2* Dickkopf-related protein 2, *Fn* fibrin, *Hotair* HOX Transcript Antisense RNA, *HOXA3* Homeobox A3, *HSC70* heat shock cognate protein 70, *HSP70* heat shock protein70, *IL-6* interleukin 6, *LPS/TLR4* lipopolysaccharide*/*Toll-like receptor 4, *LUST* Luca-15-specific transcript, *LYVE-1* lymphatic vessel endothelial hyaluronan receptor 1, *mascRNA* MALAT1-associated small cytoplasmic RNA, *Malat1* metastasis-associated lung adenocarcinoma transcript 1, *MEG9* maternally expressed 9, *MMP-2* matrix metallopeptidases*-2*, *MHC I and II* major histocompatibility complex I and II, *NEAT1* nuclear enriched abundant transcript 1, *NF-κB* nuclear factor kappa-light-chain-enhancer of activated B cells, *OA* osteoarthritis, *PRINS* psoriasis-susceptibility related RNA gene induced by stress, *PsA* psoriatic arthritis, *RA* rheumatoid arthritis, *Rank* receptor activator of nuclear factor kappa-B, *RGS2* regulator Of G protein signaling 2, *SNHG4* small nucleolar RNA host gene 4, *Spα* surface plasmon alfa, *TNF* tumor necrosis factor, *TUG1* taurine upregulated gene 1

### MSC-derived exosomes and OA

There has been ever-increasing interest in the clinical application of MSCs for a variety of diseases in recent years. MSCs have been widely studied for their potential to treat joint damage and OA [27, 79–83]. The MSCs have usually been isolated from synovium [84], bone marrow [85], and adipose tissue [21]. Researchers have assessed the effectiveness of MSCs in restoration of damaged tissue function or alleviate disease symptoms in OA or cartilage damage [86, 87]. For example, the safety and tolerability over the medium-term (~ 5 years) of intra-articular injection of MSCs has been shown for the treatment of knee in the OA in phase I and II clinical trials. This success has led to the reduction of pain and enhancement of joint performance and improved quality of cartilage repair [88–90].

It has been revealed that MSC-derived exosomes could protect cartilage and bone from degradation in OA by increasing the expression of chondrocyte markers like type II collagen and aggrecan, reducing catabolic markers such as MMP-13 and ADAMTS5, decreasing inflammatory markers (iNOS), protecting chondrocytes from apoptosis, and blocking of macrophage activation [2]. Furthermore, it has been exhibited that MSC-derived exosomes could attenuate OA by stimulation of chondrocyte migration and proliferation [38].

The motivation for transplantation of MSC for treatment of joint diseases is based on their potential for multi-lineage differentiation into mesenchymal cell types (e.g., bone, cartilage, and fat tissues) which are all essential for musculoskeletal repair. Of course, treatment with allogeneic MSC is relied on the lack of evoking an immune response [90]. Nevertheless, numerous researchers have found that in spite of the functional enhancement (or even the regeneration of joint tissue) which was observed following transplantation of MSCs into diseased joints, their engraftment and subsequent differentiation into the desirable cell types only occurred only rarely [91]. The paracrine function of the MSCs has been suggested to explain these observations, with the hypothesis that MSCs principally execute their therapeutic effect through the secretion of trophic factors that improve regeneration and decrease inflammation [92]. In the next section, the promising role of MSC-derived exosomes in the mitigation of inflammation in the OA is discussed in detail.

### MSC-derived exosomes and their anti-inflammatory effects in OA

Although OA is commonly regarded as a degenerative disease, recent work has proposed that low-grade inflammatory processes are able to induce disease symptoms and promote the progression of disease [93]. Hence, immunomodulatory function of MSCs should be further considered. MSCs are able to promote the regeneration of joint components through their two secretory functions as follows:

- Anti-inflammatory factors. MSCs may downregulate inflammatory signals in osteoarthritic cartilage, by secretion of interleukin (IL)-1β, IL-6, IL-8, matrix metalloproteinase (MMP)-1, and MMP-13 [94, 95].
- Trophic factors. These are molecules that give rise to cell proliferation, decrease formation of scar tissue, and trigger the repair of endogenous cartilage, such as epithelial growth factor (EGF), insulin-like growth factor (IGF)-1, basic fibroblast growth factor (bFGF), transforming growth factor (TGF)-β, and vascular endothelial growth factor (VEGF) [94, 96].

Although there is much evidence for the ability of MSCs to heal damage to joints, and to treat OA, there are several problems with the approach of direct cell transplantation, such as the poor survival of the cells after injection, the inability to predict lasting improvements in cell behavior and cell-cell interactions, and problems in maintaining an adequate storage bank of cells to allow off-the-shelf treatment [97]. The suitability of donors may be another important challenge, because it was found that MSCs isolated from old or otherwise unhealthy donors led to decreased performance and proliferation [98]. Moreover, the induction of senescence, loss of proliferative potential, and reduced capacity for differentiation (particularly beyond 10–20 population doublings) have been attributed to prolonged ex vivo cell expansion of MSCs before transplantation [98]. Because of their genetic programming to undergo calcification after chondrogenic induction as part of the normal process of endochondral ossification, there are problems in maintaining the cartilage phenotype in differentiated MSCs and preventing them from expanding towards the osteogenic phenotype [99]. Moreover, MSCs are sensitive to certain “environmentally responsive” factors and undergo specific behavioral modification in response to minute traces of unknown substances in the microenvironment [96]. Although this responsiveness is frequently utilized in regenerative medicine, it can have a negative impact on the MSC response in a diseased joint environment. For example, reports have demonstrated that human adipose tissue-derived MSCs can switch to a pro-inflammatory secretome, when treated with tumor necrosis factor (TNF), and can then play a role in augmenting the inflammatory response [100]. Therefore, current studies have focused on testing the secretory products of MSCs, including MSC-derived exosomes in experimental models of joint damage and OA, rather than transplanting the cells themselves.

There are many similarities between the biological effects of stem cell-derived EVs and the intact stem cells themselves. However, stem cell-derived EVs have advantages (e.g., small dimensions, low immunogenicity, and elimination of procedural issues related to direct cell injection). EVs were first described as “platelet dust” in the 1960s [101] and have been intensely investigated by researchers during the past decades. EVs are now realized to be powerful intercellular messengers that not only can mediate pathological processes, but can also maintain tissue homeostasis, and can modulate physiological functions in a variety of therapeutic approaches [102, 103]. They have been studied as anti-tumor treatments, to improve vaccination against pathogens, as immunomodulatory treatments, as drug delivery vectors, and to improve regenerative therapies [104]. Although several studies have been conducted on the use of stem cell-derived EVs as a treatment for joint damage and OA, there is still a lack of hard evidence [23, 58]. However, there are a few studies on this emerging and novel topic, suggesting the powerful ability of stem cell-derived EVs to improve joint repair and protect joints from degeneration after damage (Table 2).

**Table 2:** Exosome derived from various types of stem cells and arthritis

Cargo	Composition of cargo	Type of arthritis	Type of stem cell	Model	Function	Ref
Protein	CD9, CD63, and TSG101	OA	SMMSC and iMSCs	Mice	Induce the chondrocyte migration and proliferation	[38]
Non-coding RNA	miR-92a-3p	OA	MSCs	Mice	Modulate the development of cartilage and homeostasis via direct targeting and inhibition of WNT5A	[37]
	miR-320c	OA	hBMSCs	In vitro	Downregulate the MMP13 and up regulate SOX9)expression	[105]
	miR-140-5p	OA	SMSCs	Rat	Improve the proliferation and migration of articular chondrocytes and prevent OA	[90]
	miR-150-5p	RA	MSCs	Mice	Decrease joint destruction though inhibiting the synoviocyte hyperplasia and angiogenesis	[106]
	lncRNA-KLF3-AS1	OA	MSCs	Rat	Inhibit IL-1β-induced apoptosis of chondrocytes	[107]
Not mentioned	Not mentioned	OA	MSCs	Rabbit	Inhibit the phosphorylation of p38 and ERK and induces the phosphorylation of Akt	[108]
Not mentioned	Not mentioned	OA	MSCs	Rat	Improve the synthesis of s-GAGOmpedes by IL-1β, and inhibits the IL-1β-induced nitric oxide and MMP13 production	[109]
Not mentioned	Not mentioned	OA	AD-MSCs	In vitro	Decrease the generation of inflammatory mediators such as TNF-*α*, IL-6, PGE2, and NO and improve the generation of the anti-inflammatory cytokine IL-10	[110]
Not mentioned	Not mentioned	OA	AD-MSCs	In vitro	Downregulate the SABG activity and. Reduce the generation of inflammatory mediators.	[111]
Not mentioned	Not mentioned	OA	BM-MSCs	Mice	Induce the type II collagen, and aggrecan expression, inhibit the expression of the MMP-13, ADAMTS5, and iNOS	[2]
Not mentioned	Not mentioned	OA	ESC-MSCs	Mice	Augment the synthesis of collagen type II and reduce the expression of ADAMTS5 in the presence of IL-1β	[36]
Not mentioned	Not mentioned	Collagen-Induced Arthritis	MSCs	Mice	Anti-inflammatory role on T and B-lymphocytes	[112]
Not mentioned	Not mentioned	Antigen-induced synovitis	MSCs	Pig	Reduce the synovial lymphocytes together with a down modulation of TNF-α transcripts	[113]

*ADAMTS5* A disintegrin and metalloproteinase with thrombospondin motifs 5, *AD-MSCs* adipose-derived MSCs, *ESCs* embryonic stem cells, *ERK* extracellular-signal-regulated kinase, *KLF3-AS1* KLF3 antisense RNA 1, *IL-1β* Interleukin 1 beta, *iMSCs* iPSC-derived MSCs, *iNOS* inducible *nitric oxide synthase*, *hBM-MSCs* human bone marrow-MSCs, *MMP13* matrix metalloproteinase-13, *MSCs* mesenchymal stem cells, *NO* nitric oxide, *OA* osteoarthritis, *SMMSC* synovial membrane, *PGE2* prostaglandin E2, *s-GAG* sulfated glycosaminoglycan, *SOX9* SRY-Box 9, *RA* rheumatoid arthritis, *TNF*α tumor necrosis factor alpha, *TSG101* tumor susceptibility gene 101, *WNT5A* Wnt Family Member 5A

Zhu et al. carried out a comparative study between exosomes derived from synovial membrane MSCs (SMMSC-Exos) and exosomes derived from induced pluripotent stem cell-derived MSCs (iMSC-Exos) to treat OA [38]. These researchers showed that the diameter of both SMMSC-Exos and iMSC-Exos was between 50 and 150 nm. These exosomes expressed TSG101, CD9, and CD63. Injection of both SMMSC-Exos and iMSC-Exos into a murine OA model reduced symptoms of OA; however, a greater therapeutic impact was achieved with iMSC-Exos compared to SMMSC-Exos. Both SMMSC-Exos and iMSC-Exos triggered the proliferation and migration of chondrocytes. Overall, they showed a higher therapeutic impact of iMSC-Exos in comparison with SMMSC-Exos [38, 114]. Since autologous iMSCs can be more readily obtained, they may provide a new treatment procedure for OA [38].

Zhang et al. evaluated the contribution of MSC exosomes to regulate the inflammatory response, nociceptive behavior, condylar cartilage, and subchondral bone preservation in an immunocompetent rat model of temporomandibular joint osteoarthritis (TMJ-OA) [109]. These researchers showed that there was an initial inhibition of pain and degeneration with decreased inflammation, accompanied by a strong proliferation and gradual enhancement in matrix expression and the architecture of the subchondral bone, which demonstrated an overall joint repair and reconstruction. Chondrocyte culture can be used to assess cellular activities during exosome-mediated joint repair, measuring adenosine signaling and the activity of AKT, ERK, and AMPK. MSC-derived exosomes increased s-GAG synthesis that had been inhibited by IL-1β, and reduced IL-1β-induced nitric oxide and MMP13 production. Inhibitors of adenosine receptor activation and AKT, ERK, and AMPK phosphorylation partly reduced these effects. Overall, Zhang et al. observed increased TMJ restoration and reconstruction in OA, via a mechanism mediated by MSC exosomes. This mechanism involved several cellular processes involving repair of the matrix and general joint homeostasis. The researchers suggested this was a cell-free, ready-to-use exosome-based therapy to treat TMJ pain and degeneration [109].

### Therapeutic potentials of MSC-derived exosomes from different cell origins in OA

The cargo of exosomes derived from various cell types is highly heterogeneous and comprised of different biological molecules such as proteins, nucleic acids, and lipids that not only show the cell origin of exosomes, but also reflect the pathological or physiological conditions of the cell of origin [115, 116].

MSC-derived exosomes take part in intercellular communications and also deliver various miRNAs, mRNAs, and proteins into recipient cells, in a similar manner to exosomes derived from other cells [97]. Up to now, more than 150 miRNAs and 850 unique gene products have been recognized in MSC-derived exosomes [117, 118]. Prior studies have shown that, depending on the MSC source, the function and phenotype of MSC-derived exosomes may vary [119]. By utilizing RNA sequencing and comparative analysis, it has been shown that the content of exosomes derived from MSCs of human adipose tissue and bone marrow has significant differences in their composition, like certain tRNA species (Nanog, POU5F1A/B, and Sox2 expression), which seems to be correlated with the differentiation status of MSCs and their cell of origin [119, 120]. The therapeutic properties of human MSCs derived from adipose tissues, bone marrow, and endometrium have been recently compared in an experimental model of myocardial infarction [121]. The findings revealed the superior cardioprotection with endometrial MSCs compared to adipose and bone marrow-derived MSCs [121]. These data suggest that intrinsic differences among exosomes derived from MSCs isolated from different cellular sources should be considered as these differences may have profound impact on therapeutic outcomes [121].

The degeneration of cartilage tissue during OA progression is caused by chronic inflammation. Adipose tissue-derived MSCs (AD-MSCs) have the potential to blunt degenerative and inflammatory processes in OA. These cells also showed a paracrine effect on chondrocytes. Tofiño-Vian et al. have revealed that EVs isolated from human AD-MSCs exerted chondroprotective functions through multiple mechanisms such as reduced the production of inflammatory mediators (e.g., TNF-α, IL-6, PGE2 and NO), decreased the release of MMP activity, and enhanced the production of the anti-inflammatory cytokine IL-10 [110]. The authors concluded that AD-MSC extracellular vesicles can be employed for the development of new therapeutic approaches in joint conditions.

Researchers have comparatively evaluated the function of exosomes or microvesicles/microparticles (MPs) in OA [2]. These researchers showed that there is no significant difference between function of exosomes and MPs derived from BM-MSCs. They also found that treatment with both exosomes and MPs derived from BM-MSCs can not only restore the expression of mature articular chondrocytes markers (e.g., type II collagen and aggrecan), but also decrease the expression of catabolic (e.g., MMP-13, ADAMTS5) and inflammatory (e.g., iNOS) markers in a dose-dependent manner. Intriguingly, the highest dose of BM-MSC-derived MPs or exosomes could not only reverse the OA phenotype of chondrocytes but also could modulate anabolic and catabolic chondrocyte markers to a similar extent as BM-MSCs. Another important finding was that preactivation of BM-MSCs with TGFβ3 can improve the effectiveness of both exosomes and MPs. Although the differences were relatively small in comparison with non-preactivated BM-MSCs, the gene expression pattern was significantly different in the MPs or exosomes derived from pre-activated BM-MSCs [2]. In addition, researchers have shown that BM-MSCs may contribute to mediate an anti-fibrotic function [122]. They speculated that TGFβ3 preactivation of BM-MSCs could trigger their anti-fibroblastic and pro-chondrogenic function via the release of factors which could be transferred by exosomes and MPs. They proved the beneficial effects of MPs and exosomes pathogenic proliferation of fibroblast and skin fibrosis. In aggregate, these studies indicate that the therapeutic potential of MSCs and their derivative exosomes. These studies further emphasized that therapeutic potential of MSC-derived exosomes could largely vary based on their content composition and cell of origin in different pathological and physiological conditions and should be considered for the therapeutic applications. Recompositioning of exosome content through ex vivo pretreatment and/or preactivation of MSCs with different compounds (e.g., TGFβ3) would be also another interesting therapeutic approach to maximize anti-OA potential of exosomes.

## Conclusions

Exosomes carry out many different functions in organisms that include repair of tissue injuries, regulation of immune response, and inhibition of inflammation. Due to their ability to repair damaged tissues, MSC-derived exosomes have been widely studied in regenerative medicine. The improvement in tissue homeostasis caused by MSCs can be produced by cell-to-cell direct interaction and also by secretion of soluble factors. Exosomes are a kind of soluble biological mediator isolated from MSCs culture media in vitro. MSC-derived exosomes are generated under both pathological and physiological conditions. They are primary mediators of intercellular communications by transferring mRNAs, lipids, siRNA, proteins, miRNAs, and ribosomal RNAs to adjacent cells or remote cells. Different disease models have been studied in MSC-derived exosome experiments. Findings have demonstrated a similarity of function between MSC-derived exosomes and intact MSCs themselves. This review has presented the evidence for MSC-derived exosomes as a new approach to cell-free treatment of OA and joint damage. It is clear that numerous additional investigations must be performed to prove the effectiveness and feasibility of MSC-derived exosomes in the therapy of OA in patients.

## Data Availability

The primary data for this study is available from the authors on direct request.

## Abbreviations

AD-MSCs: Adipose tissue-derived mesenchymal stem cells
ALG-2: Apoptosis-linked gene 2
ALIX: ALG-2-interacting protein X
bFGF: Basic fibroblast growth factor
BM-MSCs: Bone marrow-derived mesenchymal stem cells
DMM: Destabilizing a medial meniscus
dsDNAs: Ribosomal RNAs
EGF: Epithelial growth factor
ESCRT: Endosomal sorting complexes required for transport
EVs: Extracellular vesicles
HEK: Human embryonic kidney cell line
IGF-1: Insulin-like growth factor1
KEGG: Kyoto Encyclopedia of Genes and Genomes
LD-Exo: Lower density exosomes
LncRNA: Long noncoding RNAs
MMP: Matrix metalloproteinase
MPs: Microparticles
MSCs: Mesenchymal stem cells
MVB: Multi-vesicular bodies
NO: Nitric oxide
NSAID: Non-steroid anti-inflammatory drugs
OA: Osteoarthritis
PBMCs: Peripheral blood mononuclear cells
PGE2: Prostaglandin E2
PLD2: Phospholipase D2
Rab: Ras-related gtp-binding protein
rRNAs: Ribosomal RNAs
SLC38A1: SFBs: Synovial fibroblasts; solute carrier family 38 member 1
TGF- β: Transforming growth factor-beta
TMJ-OA: Temporomandibular joint osteoarthritis
TNF: Tumor necrosis factor
tRNAs: Transfer RNAs
VEGF: Vascular endothelial growth factor
VPS4: Vacuolar protein sorting-associated protein
